# Psychometric properties of EQ-5D-5L for use in patients with Graves’ disease

**DOI:** 10.1186/s12955-023-02177-z

**Published:** 2023-08-15

**Authors:** Xiaodong Liu, Wendy WL Chan, Eric HM Tang, Alex HY Suen, Matrix MH Fung, Yu Cho Woo, Shirley YW Liu, Cindy LK Lam, Nan Luo, Carlos KH Wong, Brian HH Lang

**Affiliations:** 1 Department of Surgery, School of Clinical Medicine, Li Ka Shing Faculty of Medicine, University of Hong Kong, Hong Kong SAR, China; 2 Department of Clinical Oncology, School of Clinical Medicine, Li Ka Shing Faculty of Medicine, University of Hong Kong, Hong Kong SAR, China; 3 Department of Family Medicine and Primary Care, School of Clinical Medicine, Li Ka Shing Faculty of Medicine, University of Hong Kong, Hong Kong SAR, China; 4 Department of Pharmacology and Pharmacy, Li Ka Shing Faculty of Medicine, University of Hong Kong, Hong Kong SAR, China; 5 Department of Medicine, School of Clinical Medicine, Li Ka Shing Faculty of Medicine, University of Hong Kong, Hong Kong SAR, China; 6 Division of Endocrine Surgery, Department of Surgery, Prince of Wales Hospital, Faculty of Medicine, Chinese University of Hong Kong, Hong Kong SAR, China; 7 Saw Swee Hock School of Public Health, National University of Singapore, Singapore, Singapore; 8 Laboratory of Data Discovery for Health (D²4H), Hong Kong SAR, China

**Keywords:** EQ-5D-5 L, Psychometric properties, Reliability, Validity, Responsiveness, ThyPRO-39

## Abstract

**Background:**

The EQ-5D-5 L is a commonly used generic measure of health. This study aimed to evaluate the psychometric properties of the EQ-5D-5 L in patients with Graves’ disease (GD).

**Methods:**

A prospective cohort of patients with GD recruited at three public hospitals in Hong Kong completed the EQ-5D-5 L and ThyPRO-39 questionnaires at baseline, 1-month, and 6-month follow-ups. Convergent validity was tested by examining the Spearman correlation between EQ-5D-5 L and ThyPRO-39 scores at baseline. 1-month test-retest reliability was assessed by Intraclass Correlation Coefficient (ICC), Gwet’s Agreement Coefficient 2 (AC2), and percentage agreement. Responsiveness of EQ-5D-5 L index and EQ-VAS scores was assessed using effect size statistics (standardized effect size [SES] and standardized response mean [SRM]).

**Results:**

Of 125 recruited patients, 101 (80.8%) and 100 (80.0%) patients were followed up at 1- and 6-month, respectively. For convergent validity, there was a moderate negative correlation between EQ-5D-5 L index or EQ-VAS score and ThyPRO-39 overall QoL-impact score (-0.350, -0.451), between EQ-VAS score and composite score (-0.483), and strong negative correlation between EQ-5D-5 L index score and composite score (-0.567). The Gwet’s AC2 and percentage agreement were the highest in self-care (0.964 and 0.967), followed by mobility (0.952 and 0.962), usual activities (0.934 and 0.948), pain/discomfort (0.801 and 0.887), and anxiety/depression (0.788 and 0.882). The ICC for the EQ-5D-5 L index and the EQ-VAS was 0.707 and 0.700. For patients who reported having ‘worsened’ health at 6-month follow-up, the SES and SRM were − 0.66 and − 0.42 for EQ-5D-5 L index and − 1.15 and − 1.00 for EQ-VAS, respectively.

**Conclusions:**

The EQ-5D-5 L demonstrated convergent validity, test-retest reliability, and responsiveness to worsened health status among patients with GD.

**Supplementary Information:**

The online version contains supplementary material available at 10.1186/s12955-023-02177-z.

## Introduction

Graves’ disease (GD) is the most common cause of hyperthyroidism, which is caused by the production of autoantibodies against the thyrotropin receptor (TSH-R), accordingly stimulating the autonomous production of thyroid hormones [1]. According to the previous study, the overall incidence rates of childhood GD in Hong Kong were 3.2 and 6.5 per 100,000 person-years for the two periods 1989-93 and 1994-98, respectively [2]. Anti-thyroid drugs (ATD), one of the most commonly used treatments for GD, are effective in normalizing thyroid hormone levels within a short period [3, 4]. Radioactive iodine (RAI) destroys the follicular cell and gradually leads to the control of thyrotoxicosis [5]. Definitive treatment of thyroidectomy has long-lasting effects on developing hypothyroidism after removing the thyroid glands and requires thyroid hormone supplementation [6]. It has been reported that a substantial proportion of patients have altered mental health issues even after successful therapy for GD [7]. In addition to the mechanism of hyperthyroidism, Graves’ autoimmune process, and ophthalmopathy may also be involved [7].

Assessment of GD patients’ health-related quality of life (HRQoL) is important for determining the outcomes of treatments. Both specific and generic questionnaires have been used in the measurement of HRQoL in patients with GD [8–12]. The study by TÖrring et al. using the Thyroid-Related Patient-Reported Outcome (ThyPRO-39) questionnaire and 36-item Short Form Health Status survey observed lower HRQoL in GD patients receiving RAI compared to those treated with ATD or thyroidectomy [11]. Another study by Mangelen et al. using a thyroid-disease-specific questionnaire showed that the HRQoL was significantly better in RAI group compared to ATD group in three domains of goiter symptoms, emotional susceptibility, and impaired daily life [12]. Previous studies have also revealed that persistent symptoms of Graves’ ophthalmopathy (GO) and the treatments of thyroid diseases undermined the vital quality of life [9, 13].

The EQ-5D-5 L questionnaire is a generic preference-based measure used to assess HRQoL, which can be applied to a broad range of populations and settings [14]. The EQ-5D-5 L’s descriptive system contains five domains with one item per domain. Responses to these items can be converted into health utility scores using preference-based weights. To our knowledge, there is no study assessing the HRQoL with EQ-5D-5 L in GD patients. Although EQ-5D-5 L has been previously used as an outcome measure in patients with benign thyroid nodules [15, 16], little is known about the psychometric properties of the instrument used in patients with GD.

For evaluating health outcomes and cost-effectiveness, the utility instrument must demonstrate good internationally agreed measurement properties. Therefore, it is essential to validate the ability of instruments to the assessment of utility in GD patients. This study aimed to evaluate the psychometric properties, including reliability, validity, and responsiveness, of the EQ-5D-5 L questionnaire for patients with GD.

## Methods

### Study population and source of data

For study design, the COSMIN Study Design checklist suggests a sample size of at least 100, which is considered to be of ‘very good’ quality for validity, reliability, known-group comparisons, and responsiveness [17]. To account for a non-completion and withdrawal rate of 20%, this study recruited a prospective cohort of 125 patients with relapsed GD using a convenience sampling method at three public hospitals under the Hong Kong Hospital Authority between June 2020 and September 2021. Eligible patients were identified as those who were diagnosed with relapsed GD, aged 18 years or older, and able to read and understand Chinese or English questionnaires. The exclusion criteria were cognitive impairment or pregnancy. After obtaining informed consent, patients were invited to self-complete the EQ-5D-5 L and ThyPRO-39 questionnaires at baseline. Then, patients were asked to self-complete the questionnaires online at 1-month and 6-month follow-ups. At the end of the 6-month follow-up survey, following the administration of EQ-5D-5 L, patients were asked to assess their overall health condition compared to that at baseline. Given the mandatory setting of the survey questions, there was no missing information for patients who finished the health outcome questionnaires at baseline and follow-ups. The questionnaire items were not repeated for each follow-up in our survey, and there were no irrational answers detected. Socio-demographic and clinical data, including patients’ disease duration, treatment, comorbidity, and laboratory test parameters of thyroid-stimulating hormone (TSH) and free thyroxine (FT4), were extracted from the electronic database of the Hospital Authority (Hong Kong Clinical Data Analysis and Reporting System (CDARS)). This study has been approved by the local institutional review board.

### Study instruments

The EQ-5D-5 L developed by the EuroQol Group is a generic preference-based measure, which assesses patients’ self-reported health in mobility, self-care, usual activities, pain/discomfort, and anxiety/depression each with five response levels (no problems, slight problems, moderate problems, severe problems, and extreme problems) [14]. This instrument has been validated for use in the population of Hong Kong [18, 19]. Accordingly, the EQ-5D-5 L data collected in this study were converted to index scores using the Hong Kong-specific value set in this study [20]. The EQ-VAS is a 20 cm vertical visual analogue ranging from 0 (worst imaginable health) to 100 (best imaginable health), on which patients are asked to choose a number as a comprehensive assessment of their health status on the way of the survey.

The ThyPRO questionnaire developed by Watt and colleagues is a well-validated instrument for measuring thyroid-related quality of life [21]. The shorter version namely ThyPRO-39 generates 13 scales: goiter symptoms, hyper- and hypothyroid symptoms, eye symptoms, tiredness, cognitive impairment, anxiety, depressivity, emotional susceptibility, impairment in social and daily life, cosmetic complaints, and the overall QoL-impact scale. The validity of ThyPRO-39 used in Chinese patients with benign thyroid diseases has been identified by previous study [22]. The ThyPRO-39 scores range from 0 to 100, in which a greater score indicates worsening HRQoL.

### Statistical analysis

Baseline characteristics of recruited patients were described as frequencies and percentages for categorical variables and mean ± standard deviations (SD) for continuous variables. The comparison was conducted for baseline characteristics between patients who completed and lost to the 6-month follow-up to assess selection bias due to loss to follow-up. The proportion of patients giving the highest and lowest response levels were calculated to assess whether there were any floor and ceiling effects. Presence of floor or ceiling effects was considered if more than 15% of patients reported the worst or the best responses. The mean (SD) values of the EQ-5D-5 L index and EQ-VAS scores were calculated at baseline, 1-month, and 6-month follow-up.

Convergent validity was assessed using the Spearman correlation coefficient between EQ-5D-5 L index and EQ-VAS scores and ThyPRO-39 overall QoL-impact and composite scores. A coefficient value of > 0.5 was considered as strong, 0.35 to 0.5 as moderate, and 0.2 to 0.35 as a weak correlation [23]. We hypothesized that EQ-5D-5 L and EQ-VAS would be moderately or strongly correlated with the ThyPRO-39.

The timeframe for the evaluation of test-retest reliability was 1-month [24]. In our study, agreement in response levels by each dimension among patients with unchanged health conditions between baseline and 1-month follow-up was evaluated by Gwet’s agreement coefficient 2 (AC2) and percentage agreement. Gwet’s AC2 is a weighted inter-rater agreement used for ordinal variables [25]. A Gwet’s AC2 value of < 0.2 was considered as poor; 0.21 to 0.4 as fair, 0.41 to 0.6 as moderate, 0.61 to 0.8 as good, and > 0.8 as very good agreement [26]. Test-retest reliability of the EQ-5D-5 L summary index and the EQ-VAS score was calculated by Intraclass Correlation Coefficient (ICC, two-way random effects, absolute agreement, average measure). An ICC value of < 0.5 was considered as poor; 0.5 to 0.75 as moderate, 0.75 to 0.9 as good, and > 0.9 as excellent reliability. [27]

The responses assessing the health condition of patients at 6-month follow-up compared to baseline were categorized into three scenarios of health: ‘worsened’, ‘unchanged’, and ‘improved’. The mean scores between baseline and 6-month follow-up in each subgroup were compared using Wilcoxon signed-rank test. The responsiveness in EQ-5D-5L index and EQ-VAS scores in the ‘improved’ and ‘worsened’ subgroups was assessed using effect size statistics (standardized effect size [SES] and standardized response mean [SRM]). The results were interpreted as that, a SES or SRM value of 0.2 to 0.5 was considered as small, 0.5 to 0.8 as moderate, and ≥ 0.8 as large effect [28].

All statistical analyses were performed using Stata version 16.0 (StataCorp, College Station, Texas).

## Results

Table 1 shows the baseline characteristics of all recruited patients. The majority of patients were female (72.8%), aged ≤ 60 years (84.0%), and had secondary (41.6%) or higher (48.8%) education. In terms of comorbidities, 7.2%, 12.8%, and 8.0% of patients were with cardiovascular disease, hypertension, and diabetes, respectively. 15 (12.0%), 77 (61.6%), and 33 (26.4%) patients received ATD, RAI, and surgical treatment for GD, respectively. 15.2% of patients were current smokers, and 34.4% were current drinkers. More than a third of patients (38.4%) had Graves’ ophthalmopathy. Among a total of 125 GD patients recruited at baseline, 101 (80.8%) and 100 (80.0%) patients were followed up at 1 and 6 months. No statistical difference in baseline characteristics were observed between patients who completed or lost to the 6-month follow-up. (Supplemental Table 1)

**Table 1:** Baseline characteristics of patients (n = 125)

Characteristic	N (%) or mean ± SD
**Sociodemographic**	
Age, year	43.73 ± 13.83
Age group	
≤60	105 (84.0%)
>60	20 (16.0%)
Gender	
Male	34 (27.2%)
Female	91 (72.8%)
Highest education level attained	
Primary	12 (9.6%)
Secondary	52 (41.6%)
Tertiary	61 (48.8%)
Marital status	
Married	69 (55.2%)
Non-married	56 (44.8%)
Smoking	
Smoker	19 (15.2%)
Non-smoker	93 (74.4%)
Ex-smoker	13 (10.4%)
Drinking	
Drinker	43 (34.4%)
Non-drinker	69 (55.2%)
Ex-drinker	13 (10.4%)
Monthly household income	
≤HKD20,000	56 (44.8%)
>HKD20,000	45 (36.0%)
Refuse to answer	24 (19.2%)
**Clinical parameters**	
Duration of GD, month	70.48 ± 5.52
TSH, mIU/l	3.25 ± 10.12
FT4, pmol/l	21.78 ± 14.08
**Treatment**	
Anti-thyroid drugs	15 (12.0%)
Radioactive iodine	77 (61.6%)
Thyroidectomy	33 (26.4%)
**Comorbidity**	
Cardiovascular diseases	9 (7.2%)
Diabetes mellitus	10 (8.0%)
Hypertension	16 (12.8%)
Liver disease	3 (2.4%)
Chronic obstructive pulmonary disease	3 (2.4%)
Graves’ ophthalmopathy	48 (38.4%)

Notes: TSH, thyroid-stimulating hormone; FT4, free thyroxine; GD, Graves’ disease; SD, standard deviation

The mean EQ-5D-5 L index and EQ-VAS scores were estimated at baseline, 1-month, and 6-month follow-ups. Most patients reported ‘no problems’ in the self-care domain. A ceiling effect was observed in the EQ-5D-5 L index score at baseline. 28.0% and 5.6% of patients reported perfect health state for EQ-5D-5 L (11,111) and best imaginable health for EQ-VAS (100), respectively. The proportion of patients with the best response in each domain of EQ-5D-5 L was 88.0% (mobility), 94.4% (self-care), 81.6% (usual activity), 55.2% (pain/discomfort), and 46.4% (anxiety/depression), respectively. Mean (± SD) EQ-5D-5 L index and EQ-VAS scores were 0.91 ± 0.10 and 79.16 ± 13.01 at baseline, 0.88 ± 0.15 and 78.91 ± 14.50 at 1-month, and 0.90 ± 0.11 and 77.95 ± 14.76 at 6-month follow-up, respectively. (Supplemental Table 2)

The spearman’s correlation was estimated between the EQ-5D-5 L index and EQ-VAS scores and ThyPRO-39 summary scores at baseline. A moderate negative correlation was observed between EQ-5D-5 L index score and ThyPRO-39 Overall QoL-impact score (-0.350), EQ-VAS score and ThyPRO-39 overall QoL-impact score (-0.451), and EQ-VAS score and composite score (-0.483), while a strong negative correlation was observed between EQ-5D-5 L index score and ThyPRO-39 composite score (-0.567).

Table 2 shows the agreement of response levels by EQ-5D-5 L dimensions and ICC by EQ-5D-5 L index and EQ-VAS between baseline and 1-month follow-up among patients with self-reported ‘unchanged’ health status. Gwet’s AC2 was the highest in self-care (0.964), followed by mobility (0.952), usual activities (0.934), pain/discomfort (0.801), and anxiety/depression (0.788), and percent agreement was the highest in self-care (0.967), followed by mobility (0.962), usual activities (0.948), pain/discomfort (0.887), and anxiety/depression (0.882), indicating almost perfect or substantial reliability was achieved. The ICC for the EQ-5D-5 L index and the EQ-VAS respectively were fairly similar (EQ-5D-5 L index: 0.707, EQ-VAS: 0.700), showing moderate reliability.

**Table 2:** One-month test-retest reliability of EQ-5D-5 L dimensions, EQ-5D-5 L index and EQ-VAS scores (n = 64)

EQ-5D-5 L dimensions	Gwet’s AC2				Percentage agreement		
	Estimate	95% CI			Estimate	95% CI	
Mobility	0.952	0.833	1.000		0.962	0.848	1.000
Self-care	0.964	0.849	1.000		0.967	0.853	1.000
Usual activities	0.934	0.819	1.000		0.948	0.835	1.000
Pain/discomfort	0.801	0.685	0.918		0.887	0.779	0.995
Anxiety/depression	0.788	0.668	0.907		0.882	0.774	0.991
	Intraclass correlation coefficient (95% CI)						
EQ-5D-5 L index	0.707 (0.495,0.831)						
EQ-VAS	0.700 (0.483,0.826)						

Notes: Gwet’s AC2, Gwet’s agreement coefficient 2; CI, confidence interval; EQ-5D-5 L, EQ-5D Five-Level; VAS, visual analogue scale

Table 3 shows the responsiveness in the EQ-5D-5 L index and EQ-VAS at the 6-month follow-up. For patients who reported ‘worsened’ health at 6-month follow-up (EQ-5D-5 L index score at baseline vs. at 6 months: 0.92 ± 0.08 vs. 0.87 ± 0.10, *P* = 0.027; EQ-VAS score at baseline vs. at 6 months: 83.10 ± 9.42 vs. 72.29 ± 15.58, *P* < 0.001), SES and SRM were − 0.66 and − 0.42 for EQ-5D-5 L index, and − 1.15 and − 1.00 for EQ-VAS. In patients with ‘improved’ health (EQ-5D-5 L index at baseline vs. at 6 months: 0.92 ± 0.11 vs. 0.90 ± 0.14, *P* = 0.283; EQ-VAS at baseline vs. at 6 months: 78.12 ± 14.34 vs. 80.83 ± 13.90, *P* = 0.257), SES and SRM were − 0.16 and − 0.17 for EQ-5D-5 L index, and 0.19 and 0.20 for EQ-VAS.

**Table 3:** Responsiveness parameters at 6-month follow-up in EQ-5D-5 L index and EQ-VAS among patients with Graves’ disease

	EQ-5D-5 L Index score			EQ-VAS score		
	Worsened (n = 21)	Unchanged (n = 38)	Improved (n = 41)	Worsened (n = 21)	Unchanged (n = 38)	Improved (n = 41)
Mean (SD)						
baseline	0.92 (0.08)	0.93 (0.08)	0.92 (0.11)	83.10 (9.42)	78.95 (12.74)	78.12 (14.34)
6-month	0.87 (0.10)	0.92 (0.08)	0.90 (0.14)	72.29 (15.58)	77.97 (14.69)	80.83 (13.90)
change	-0.05 (0.12)	-0.01 (0.09)	-0.02 (0.10)	-10.81 (10.76)	-0.97 (13.85)	2.71 (13.27)
*P*-value	0.027*	0.358	0.283	< 0.001*	0.971	0.257
SES (95%CI)	-0.66 (-1.30,0.06)	-	-0.16 (-0.43,0.13)	-1.15 (-1.69, -0.75)	-	0.19 (-0.07,0.47)
SRM (95%CI)	-0.42 (-0.84,0.04)	-	-0.17 (-0.48,0.14)	-1.00 (-1.48, -0.66)	-	0.20 (-0.08,0.50)

Notes: CI, confidence interval; EQ-5D-5 L, EQ-5D Five-Level; VAS, visual analogue scale; SD = standard deviation; SES = standardized effect size; SRM = standardized response mean. Changes were calculated by subtracting baseline scores from 6-month follow-up scores, * P < 0.05 by Wilcoxon signed-rank test to compare the mean scores at baseline and at 6 months

## Discussion

To our best knowledge, this prospective cohort study is the first research to evaluate the psychometric properties of EQ-5D-5 L used in patients with GD. Results of this study indicated that EQ-5D-5 L demonstrated good reliability and convergent validity, and was responsive to changes in health outcomes over time. This study provided evidence supporting the use of the EQ-5D-5 L in assessing the HRQoL for GD patients.

The good test-retest reliability of EQ-5D-5 L showed in our study was consistent with the findings of previous studies. The study by Long et al. using the online-based questionnaire reported that Gwet’s AC ranged from 0.64 to 0.97 for EQ-5D-5 L dimensions, and the ICC ranged from 0.73 to 0.84 for the EQ-5D-5 L summary index and from 0.61 to 0.68 for EQ-VAS among the general population in Italy, the Netherlands, and the United Kingdom [29]. The study by Seng et al. supported EQ-5D-5 L as a valid and reliable instrument for assessing HRQoL among patients with axial spondyloarthritis in Singapore [30]. Similarly, in our study, the high Gwet’s AC2 value for the EQ-5D-5 L dimensions indicated almost perfect or substantial reliability and the ICC for the EQ-5D-5 L index and the EQ-VAS showed moderate reliability. Therefore, our study confirmed the good reliability of EQ-5D-5 L used for GD patients.

For the evaluation of convergent validity of the utility instrument, this study showed a moderate to strong correlation between ThyPRO-39 overall-impact or composite scores and EQ-5D-5 L index or EQ-VAS scores. The good convergent validity of EQ-5D-5 L supported in this study was previously demonstrated in the general and other patient populations [31–33]. Although EQ-5D-5 L describes patients’ quality of life in five dimensions, some variations exist in other domains among patients suffering from Graves’ hyperthyroidism, and the disease-specific instrument (e.g., ThyPRO-39) is needed. The moderate to good correlation between EQ-5D-5 L and ThyPRO-39 in this study indicated the need to utilize disease-specific and generic instruments to assess the quality of life among patients with GD.

In our study, the effect sizes estimated by SES and SRM for changes after 6 months of treatment were large for EQ-5D-5 L index and EQ-VAS scores among patients with worsened health conditions, suggesting that the EQ-5D-5 L was capable of identifying minimal changes in the subgroup of patients with health deterioration. However, the EQ-5D-5 L might not be responsive in patients who had improved health, partly due to high ceiling effects at baseline and small sample size. The magnitude of negative changes observed in patients who self-reported worsened health was a reduction of 0.05. This is consistent with a previous study reporting a summarized mean ± SD value of 0.058 ± 0.005 for the minimal clinically important difference of EQ-5D-5 L [34]. Further investigations are required to determine whether such a magnitude of change in the EQ-5D-5 L score is of meaningful value.

It has been reported that respondents will give more positive and socially desirable responses in the face-to-face interview, while those surveyed in web mode may provide fewer positive responses [35, 36]. Although online survey mode might decrease the willingness of subjects to finish the follow-up questionnaires, a relatively high completion rate (80%) was achieved at the 6-month follow-up in this study. Our study showed that 28.0% of patients self-reported no problems in all five dimensions, indicating a ceiling effect for the EQ-5D-5 L index score at baseline. This is a concern because it means the EQ-5D-5 L index score is unable to detect any improvement experienced by those patients. Nevertheless, our results were consistent with the findings of previous studies that EQ-5D-5 L might be limited by ceiling effects [32, 37, 38].

There are some limitations to this study. First, loss to follow-up might limit the findings when evaluating responsiveness. Although 80% of recruited patients completed follow-up at 6 months, incomplete follow-up might bias the results due to the loss of subjects. However, the impact of selection bias due to loss to follow-up was minimal because there was no statistical difference in baseline characteristics between patients who completed follow-up questionnaires and those who were lost to follow-up (Supplemental Table 1). Second, the small sample size might lead to wide confidence intervals and unreliable results. Therefore, the responsiveness results generated in the worsened group with the small sample size should be treated as preliminary results. Future studies with larger sample size should be conducted to assess the responsiveness among this group of patients. Additionally, our prospective cohort study was conducted among patients sampled from the endocrinology and surgical outpatient clinics of three public hospitals in Hong Kong, which might limit the generalizability of our findings.

In conclusion, our prospective cohort study supported the convergent validity and reliability of EQ-5D-5 L, as well as proven responsive to worsened health status for patients with GD. Given that EQ-5D-5 L may not be responsive in GD patients who have improved health conditions, future studies with a larger sample size are needed to explore the responsiveness of EQ-5D-5 L associated with improved health states.

### Electronic supplementary material

Below is the link to the electronic supplementary material.

Supplementary Material 1

## Data Availability

The dataset used in this current study is not available to share with any other persons for any purposes except those authorized users who are permitted to analyze and research the data.
