# Supplementary material for: Psychometric properties of EQ-5D-5L for use in patients with Graves’ disease
Source: Health Qual Life Outcomes. 2023 Aug 15;21:90. doi: 10.1186/s12955-023-02177-z (PMC10426177; doi:10.1186/s12955-023-02177-z)
Supplement: Supplementary file 1 — Supplementary Material 1 [file 12955_2023_2177_MOESM1_ESM.docx]

Supplemental Table 1. Baseline characteristics of patients who completed and lost to the 6-month follow-up

| Characteristic (N (%) or  mean ± SD) | Follow-up completion  (n=100) | Loss to follow-up  (n=25) | P-value |
| --- | --- | --- | --- |
| **Sociodemographic** |  |  |  |
| Age, year | 42.70±14.25 | 47.84±11.32 | 0.097 |
| Age group |  |  | 1.000 |
| ≤60 | 84 (84.00%) | 21 (84.00%) |  |
| >60 | 16 (16.00%) | 4 (16.00%) |  |
| Gender |  |  | 0.272 |
| Male | 25 (25.00%) | 16 (64.00%) |  |
| Female | 75 (75.00%) | 9 (36.00%) |  |
| Highest education level attained |  |  | 0.062 |
| Primary | 10 (10.00%) | 2 (8.00%) |  |
| Secondary | 36 (36.00%) | 16 (64.00%) |  |
| Tertiary | 54 (54.00%) | 7 (28.00%) |  |
| Marital status |  |  | 0.315 |
| Married | 41 (41.00%) | 8 (32.00%) |  |
| Non-married | 59 (59.00%) | 16 (68.00%) |  |
| Smoking |  |  | 0.102 |
| Smoker | 78 (78.00%) | 15 (60.00%) |  |
| Non-smoker | 12 (12.00%) | 7 (28.00%) |  |
| Ex-smoker | 10 (10.00%) | 3 (12.00%) |  |
| Drinking |  |  | 0.172 |
| Drinker | 58 (58.00%) | 11 (44.00%) |  |
| Non-drinker | 33 (33.00%) | 10 (40.00%) |  |
| Ex-drinker | 9 (9.00%) | 4 (16.00%) |  |
| Monthly household income |  |  | 0.891 |
| ≤HKD20,000 | 46 (46.00%) | 10 (40.00%) |  |
| >HKD20,000 | 35 (35.00%) | 10 (40.00%) |  |
| Refuse to answer | 19 (19.00%) | 5 (20.00%) |  |
| **Clinical parameters** |  |  |  |
| Duration of GD, month | 73.72±57.49 | 93.23±63.32 | 0.167 |
| TSH, mIU/l | 2.32±7.42 | 4.10±12.94 | 0.380 |
| FT4, pmol/l | 22.32±14.43 | 20.49±13.03 | 0.565 |
| **Treatment** |  |  | 0.629 |
| Anti-thyroid drugs | 13 (13.00%) | 2 (8.00%) |  |
| Radioactive iodine | 61 (61.00%) | 16 (64.00%) |  |
| Thyroidectomy | 26 (26.00%) | 7 (28.00%) |  |
| **Comorbidity** |  |  |  |
| Cardiovascular diseases | 5 (5.00%) | 4 (16.00%) | 0.071 |
| Diabetes mellitus | 9 (9.00%) | 1 (4.00%) | 0.423 |
| Hypertension | 12 (12.00%) | 4 (16.00%) | 0.594 |
| Liver disease | 2 (2.00%) | 1 (4.00%) | 0.567 |
| Chronic obstructive pulmonary disease | 2 (2.00%) | 1 (4.00%) | 0.567 |
| Graves’ ophthalmopathy | 39 (39.00%) | 9 (36.00%) | 0.783 |

TSH, thyroid-stimulating hormone; FT4, free thyroxine; GD, Graves’ disease; SD, standard deviation. Cardiovascular diseases include coronary heart disease, heart failure, and stroke. *Signiﬁcant with P-value < 0.05 by univariate linear regression, binary logistic or multinomial logistic regression.

Supplemental Table 2. EQ-5D-5L index and EQ-VAS scores and response distributions of EQ-5D-5L dimensions

| EQ-5D-5L dimensions | No problems, n (%) | Slight problems,  n (%) | Moderate problems,  n (%) | Severe problems,  n (%) | Unable to,  n (%) |
| --- | --- | --- | --- | --- | --- |
| Mobility | 110 (88.0%) | 14 (11.2%) | 1 (0.8%) | 0 (0%) | 0 (0%) |
| Self-care | 118 (94.4%) | 6 (4.8%) | 1 (0.8%) | 0 (0%) | 0 (0%) |
| Usual activities | 102 (81.6%) | 22 (17.6%) | 1 (0.8%) | 0 (0%) | 0 (0%) |
| Pain/discomfort | 69 (55.2%) | 48 (38.4%) | 5 (4.0%) | 3 (2.4%) | 0 (0%) |
| Anxiety/depression | 58 (46.4%) | 55 (44.0%) | 10 (8.0%) | 2 (1.6%) | 0 (0%) |
| EQ-5D-5L score | Baseline | | | 1-month | 6-month |
|  | Floor, n (%) | Ceiling, n (%) | Mean (SD) | Mean (SD) | Mean (SD) |
| EQ-5D-5L index | 0 (0%) | 35 (28.0%) | 0.91 (0.10) | 0.88±0.15 | 0.90 (0.11) |
| EQ-VAS | 0 (0%) | 7 (5.6%) | 79.16 (13.01) | 78.91±14.50 | 77.95 (14.76) |

EQ-5D-5L, EQ-5D Five-Level; VAS, visual analogue scale; SD, standard deviation
